# Appetite

**Published:** 1878-07

**Authors:** 


### APPETITE.

"*Asking for*,' that is the meaning. Who asks? *Nature;* in other words, the law of our being, the instinct of self-preservation, wisely and benevolently implanted in every living thing, whether animal, worm, or weed.

Yielding to this appetite is the preservation of all life, and health, below man; he alone exceeds it, and in consequence sickens and dies thereby, long before his prime, in countless instances.

The fact is not recognized as generally as it ought to be, that a proper attention to the "askings" of nature, not only maintains health, but is one of the safest, surest, and most permanent methods of curing disease.

It is eating without an appetite, which in many instances is the last pound which breaks the camel's back; nature had taken away the appetite, had closed the house for necessary repairs, but, in spite of her, we "*forced down some food*," and days and weeks and months of illness followed, if not cholera, cramp, colic, or sudden death.

In disease, there are few who cannot recall instances, where a person was supposed to be in a dying condition, and in the delirium of fever, or otherwise, had arisen, and gone to the pail or pitcher, and drank an enormous quantity of water, or have gone to the pantry, and eaten largely of some unusual food, and forthwith began to recover. We frequently speak of persons getting well having the *strangest kind of an appetite*, the indulgence of which reason and science would say would be fatal.

We found out many years ago, when engaged in the general practice of medicine, that when the patient was convalescing, the best general rule was, *eat not an atom you do not relish; eat anything in moderation which your appetite craves, from a pickle down to sole-leather.* Nature is like a perfect housekeeper; she knows better what is wanting in her house than anybody else can tell her. The body in disease craves that kind of food which contains the element it most needs. This is one of the most important facts in human hygiene; and yet we do not recollect to have ever seen it embodied in so many words. We have done so, to render it practical; and to make it remembered, we state a fact of recent occurrence.

Some three years ago, a daughter of James Damon, of Ches terfield, fell down a flight of stairs, bringing on an illness from which it was feared she would not recover. She did however recover, except the loss of hearing and sight. Her appetite for some weeks called for nothing but raisins and candy, and since last fall, nothing but apples were eaten. A few weeks ago she commenced eating maple buds; since which time she has nearly regained her former health and activity, and her sight and hearing are restored.

We all, perhaps, have observed that cats and other animals, when apparently ill, go out and crop a particular grass or weed. In applying these facts, let us remember to indulge this "*asking for*" of Nature, in sickness especially, in modera tion; feeling our way along by gradually increasing amounts; thus keeping on the safe side. We made this one of our ear-liest and most inflexible rules of practice.

